# Ultrasound-guided percutaneous treatment of a calcific acromioclavicular joint: A case report

**DOI:** 10.1097/MD.0000000000018645

**Published:** 2020-01-03

**Authors:** Angelo Iovane, Marco Di Gesù, Francesco Mantia, Ewan Thomas, Giuseppe Messina

**Affiliations:** a Sport and Exercise Research Unit, Department of Psychological, Pedagogical and Educational Sciences, University of Palermo; b Centro Medico Mantia, Palermo, Italy.

**Keywords:** acromioclavicular joint ;ultrasound-guided percutaneous treatment, shoulder, pain

## Abstract

**Rationale:**

Calcific tendinopathy is one of the most frequent causes of nontraumatic shoulder pain. However, intra-articular calcifications appear to be an infrequent condition. We herein report a rare case study of an intra-articular calcification of the acromioclavicular joint.

**Patient concerns:**

A 46-year-old man presented with an acute pain in the anterior superior region of the left shoulder which also radiated to the left cervical region. The man during the physical evaluation also presented severe functional limitation of the shoulder movements in all planes of motion.

**Diagnoses:**

The diagnosis was carried out through a radiographic and an echotomographic examination, highlighting the intra-articular calcific formation associated to a reactive inflammatory reaction.

**Interventions:**

An ultrasound-guided percutaneous treatment following the guidelines for calcific tendinopathy of the shoulder was carried out.

**Outcomes:**

The posttreatment was satisfactory with the disappearance of the pain and the recovery of the shoulder movements in all planes of motion.

**Lessons:**

We can affirm that the ultrasound-guided percutaneous treatment in patients with calcification of the acromion-clavicular joint represents a valid and nonpainful therapeutic treatment.

## 1 Introduction

One of the most frequent causes of nontraumatic shoulder pain (ranging between 2.7% and 42%), is represented by calcific tendinopathy.^[1]^ However, the deposition of insoluble calcium salts in the form of an acute calcification within the acromioclavicular joint is a rare condition.

The clinical diagnosis is based on the variable positivity to the different clinical tests determined by the location of the calcific deposits. The acute clinical symptomatology of an intra-articular calcification is usually characterized by invasive pain, associated with severe functional limitation of the shoulder movements with consequent severe impairment of daily life and working activities.^[2]^

In the clinical suspicion of a pathology affecting the acromioclavicular joint, the execution of a radiographic examination associated with the echotomographic examination represents the first steps of the diagnostic procedure.

The treatment of calcific formations usually involves 2 levels of treatment. The first is conservative treatment in the form of physiotherapy or exercise programs or the use of nonsteroidal anti-inflammatory drugs (NSAID). If such treatments are ineffective, corticosteroid injections of shockwave therapy may be approached.^[3]^ A 2nd level treatment may be represented by ultrasound-guided percutaneous treatment (UGPT) which has been proven to be more effective than shockwave therapy in the management of pain.^[3]^ In this study, we report a rare case of acute calcification of the acromioclavicular joint, treated through a minimally invasive conservative approach such as the UGPT, which has produced an excellent clinical result.

## 2 Case presentation

A male patient, 46 years old, 192 cm height and 85 kg weight, in January 2016 comes to our observation in relation to the appearance of an acute pain in the anterior superior region of the left shoulder which radiated to the lateral cervical and deltoideal region with an increase of the painful symptomatology at night.

The CARE reporting guidelines were adopted for the presentation of this case report.^[4]^

The patient reported not having had any direct trauma or being affected by systemic, metabolic, or endocrine disorders. The patient had practiced rest for a week, local intermittent application of ice, and oral NSAID intake under medical indication, without substantial improvements in the painful symptomatology. At our observation, the clinical evaluation of the left shoulder highlighted: lack of presence of swelling, reddening of the skin, or alterations of surface temperature; severe functional limitation during passive and active movements of the shoulder on all the range of motion planes; and intense pain during palpation of the acromioclavicular joint.

**Figure 1:**
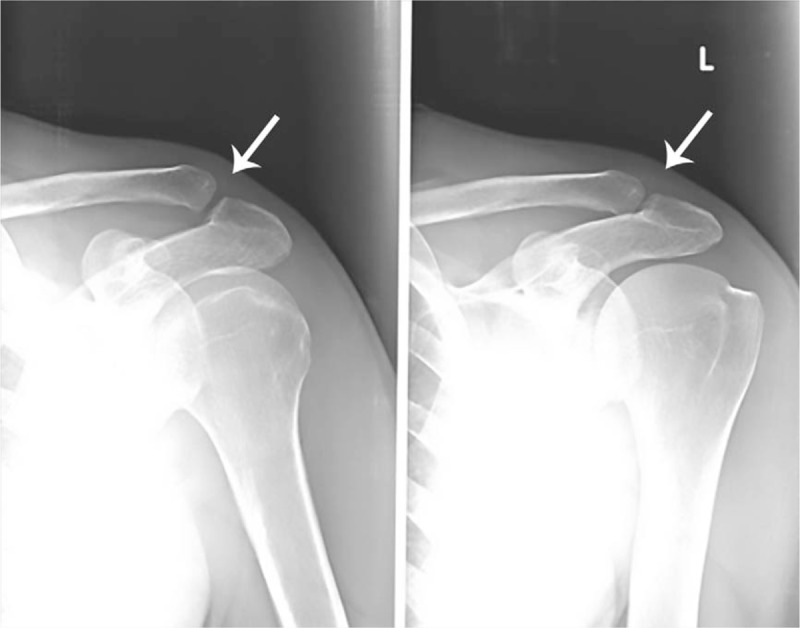
Tenuous radiopaque image in the acromioclavicular joint (arrows).

**Figure 2:**
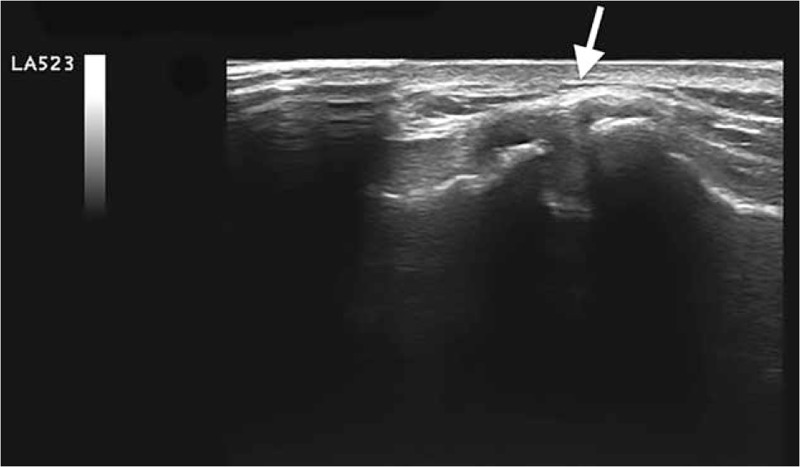
The arrow indicates the nubecular calcific formation surrounded by synovial inflammatory reaction.

The patient reported a pain value of 8/10 measured with the visual-analogical spatial (VAS) scale. A radiographic examination and an echotomographic examination of the shoulder were performed sequentially. Both instrumental examinations showed the presence of a calcific deposit inside the acromioclavicular joint (Figs. 1 and 2) with the absence of significant alterations of the bone structures of the joint and of the rotator cuff tendons, neither the presence of a reactive bursitis.

In particular, the echotomographic examination, compared to the radiographic examination, better highlighted the exact intra-articular localization of the calcification in the left acromioclavicular joint. The calcium deposit had a hyperechoic appearance, homogeneous overall in the absence of a posterior shadow, thus creating an ultrasound picture of a fluid-soft calcific image (nubecular appearance) with a maximum diameter of 12.2 mm. The calcification also appeared surrounded by diffuse and inhomogeneous hypoechogenicity in relation to the associated intra-articular inflammatory reaction (Fig. 2).

In relation to the clinical characteristics of the pathology and of the data obtained from the imaging, a diagnosis of an intra-articular calcification in reabsorption phase, of the left acromion-clavicular joint, was postulated.

A therapeutic guideline for the treatment of intra-articular calcifications of the acromion-clavicular joint has still not been described. However, based on the experience gained by our team in the treatment of calcific tendinopathy of the shoulder and the results described by other research groups for this latter type of pathology,^[1,5]^ it was decided to use the same procedure of a UGPT of a calcific tendinopathy of the shoulder. After informing the patient about the technique and the therapeutic options and after obtaining the informed consent, the UGPT of the intra-articular calcification was performed. Additionally, the patient has provided informed consent for publication of the case.

The patient was placed on an examination bed in a supine position with the left upper limb in a neutral position.

The equipment used was prepared as follows:

- an 8-mL syringe with 2% lidocaine hydrochloride,
- different 10 mL syringes with about 6 mL of physiological solution,
- a 5-mL syringe with 1 mL of methylprednisolone acetate 40 mg/mL,
- a 16-gauge (G) needle,
- a 21-G needle,
- sterile gloves,
- surgical cutaneous disinfectant (10% iodopovidone),
- bactericidal disinfectant (chlorhexidine),
- dry ice,
- ultrasound probe, and
- sterile polyurethane probe cover.

After the echotomographic evaluation to identify the exact location of the calcification and the best access route for the introduction of the needle, an accurate and wide disinfection of the shoulder, with surgical cutaneous disinfectant, and of the ultrasound probe with 70% alcohol solution, with subsequent placement of a sterile polyurethane probe cover were carried out.

**Figure 3:**
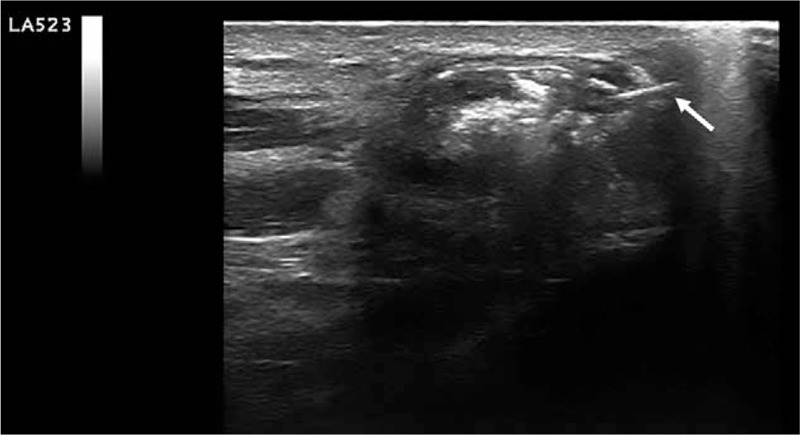
Ultrasound-guided percutaneous treatment phase. The arrow indicates the needle inserted in the context of the calcification.

The following procedure was adopted to treat the calcific formation:

1. Skin disinfection and target centering: A new skin disinfection of the region of interest with chlorhexidine was performed. The probe was then positioned in such a way as to allow an optimal visualization of the area to be treated.
2. Local anesthesia: Through the 21 G needle, 8 mL of lidocaine hydrochloride was injected, under ultrasound guidance, along the path of the needle, from the skin to the pericalcific level to anesthetize the area. The needle was then removed and 2 minutes were awaited to allow the anesthetic to make its local effect.
3. Introduction of the needle under ultrasound guidance within the calcification: A 16-G needle fitted with a 10-mL syringe partially filled with the saline solution was inserted along the short side of the probe. The needle was ecographically followed along its entire journey, from the area of its cutaneous entry to the target (intra-articular calcification) (Fig. 3). For a better visualization of the needle, it is advisable to maintain a trajectory as parallel as possible to the long axis of the probe.
4. Suction Procedure: In this phase, the alternate compression and release of the syringe plunger have been applied so that the physiological solution was injected and resuctioned inside it. This impulse was applied until the calcific amorphous material was completely aspirated inside the syringe, which will result in a modification of the color of the physiological solution due to the presence of calcium fragments. Such process may be repeated with different syringes, but without removing the needle from inside the joint, until the calcific content is totally expelled.
5. Treatment with cortisone: Without removing the needle from inside the articular location, a new 5-mL syringe was placed and 1 mL of methylprednisolone acetate was injected inside the joint.
6. Posttreatment ultrasound check: Once the needle has been extracted, the skin is again disinfected with chlorhexidine and after a few minutes, an ultrasound check of the treated part was performed to rule out any local complications. After that, the entry point was covered with sterile gauze and dry ice was applied for about 15 minutes.

The patient reported no pain throughout the treatment procedure.

After being clinically rechecked about 30 minutes after the end of the treatment, the patient was discharged with the following indications: absolute functional rest for the first 3 days; relative functional rest for further 15 days, taking care to avoid elevation and abduction movements of the joint above 90°, and avoid the use of the left upper limb to move heavy loads; ultrasound clinical examination after 3 weeks from the treatment; and application of ice packs during the first 3 hours after the intervention and intake of analgesic drugs only if needed.

No local complications after the UGPT were detected. The patient did not need local or systemic therapy for pain management after treatment.

The clinical and instrumental check carried out 3 weeks after the treatment highlighted the disappearance of nocturnal pain, a significant pain reduction, referring to a VAS scale of 1/10 and a recovery of the entire range of movement (ROM) of the left shoulder on all movement planes during both active and passive mobilizations.

**Figure 4:**
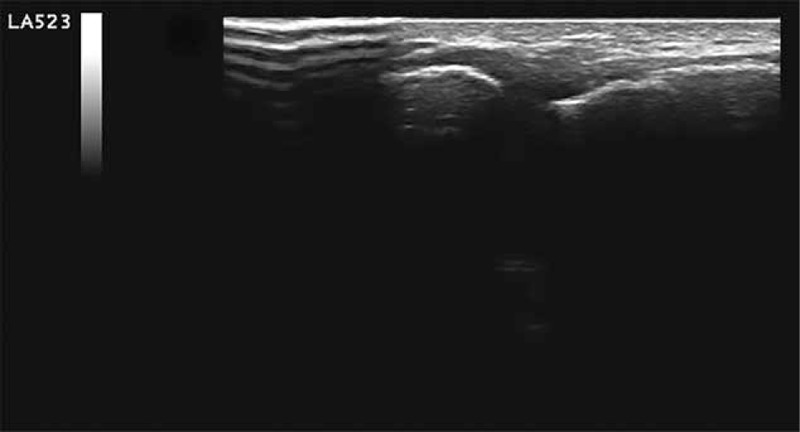
Postultrasound-guided percutaneous treatment acromioclavicular joint.

The ultrasound evaluation showed the presence of small intra-articular hyperechogenic fragments with disappearance of the intra-articular inflammatory reaction (Fig. 4).

When the patient was contacted about 3 years after the treatment, he reported that during this period he had not experienced any particular or functional disorder to the treated shoulder. He also reported that he had recommenced the sporting activities that he had discontinued previously (basketball/tennis) due to his injury. The UGPT we performed as a treatment of a calcification of the acromion-clavicular joint has been a valid and nonpainful therapeutic treatment, of rapid execution, low cost, minimally invasive, which did not show complications and of easy repeatability.

## 3 Discussion

Acromion-clavicular pain is a common condition in clinical practice. The related diseases that may be responsible for the onset of pain and functional limitation are in most cases of traumatic origin (fractures, dislocations, and hematomas)^[6]^ and to a lesser degree of atraumatic origin (arthritis, arthritis, and infections).^[7]^ Some pathological conditions may be related to an increased risk of development of soft tissue calcifications as tissue lesions, gout, chondrocalcinosis, hyperparathyroidism, renal failure, calcinosis, osteosarcoma with soft-tissue involvement, dermatomyositis, CREST syndrome, crystal deposition disease, and pyrophosphate calcium dihydrate.^[8,9]^ The formation of a deposit of calcium salts in the intra-articular site, in subjects that do not present one of the previously listed pathological conditions, is a rather infrequent event which pathogenesis is much discussed. Rui et al^[10–12]^ has hypothesized that at the base of the calcific formation, there could be an incorrect differentiation of mesenchymal stem cells in osteoblasts or chondrocytes. The material of these deposits has been identified as calcium carbonate hydroxyapatite.^[13–15]^ The radiographic method which is used to diagnose calcific formations, highlights erosive irregularities in the articular components or, as in our case, the presence of calcific formations within the joint.^[16]^ The integration with the echotomographic examination of the acromioclavicular joint can confirm the diagnosis and provide an indication of the consistency, the precise localization of the calcification and the presence of any reactive inflammatory reactions accompanied by intra-articular effusion.^[17]^

The treatment of symptomatic calcifications generally involves conservative or surgical treatments. Among the conservative treatments, minimally invasive techniques have been developed which have included the introduction of needles under ultrasound guidance as in the treatment of calcific tendinopathy of the supraspinatus tendon.^[18–20]^

Conversely, if these conservative treatments prove to be ineffective, the surgical treatment for the removal of calcified formations represents a further therapeutic possibility.^[21]^

Cases of gout, chondrocalcinosis, or deposit of calcic pyrophosphate crystals have been already described in relation to calcific intra-articular formations in the acromion-clavicular joint, which have however been secondarily related to the degenerative or metabolic changes of the affected structures.^[22–26]^

In our case, the presence of a unique calcification, classified through the echotomographic examination as nodular with a nubecular aspect, made us exclude a pathogenesis as those above mentioned.

The execution of a 2nd level investigation (computed tomography or nuclear magnetic resonance) was not considered necessary in relation to the history, the clinical examination and the data gathered from the radiographic and echotomographic examination to define the diagnosis of the calcific pathology. In consideration of the poor results obtained with the NSAID treatment previously performed by the patient, we have opted for the execution of the UGPT of the calcification in the acromion-clavicular joint. This therapeutic procedure proved to be quick and effective. The checks to which the patient has subsequently undergone showed an immediate, complete, and lasting remission of the painful symptomatology with full recovery of the shoulder function without the painful symptomatology.

## Author contributions

**Conceptualization:** Giuseppe Messina.

**Data curation:** Francesco Mantia.

**Formal analysis:** Francesco Mantia.

**Investigation:** Marco Di Gesù.

**Methodology:** Angelo Iovane.

**Supervision:** Angelo Iovane.

**Writing – original draft:** Ewan Thomas.

**Writing – review & editing:** Ewan Thomas, Giuseppe Messina.
